# Comprehensive Profile Analysis of Differentially Expressed circRNAs in Glucose Deprivation-Induced Human Nucleus Pulposus Cell Degeneration

**DOI:** 10.1155/2021/4770792

**Published:** 2021-07-02

**Authors:** Hongze Chang, Hongzhang Wang, Xiaolong Yang, Kemin You, Mingwei Jiang, Feng Cai, Yan Zhang, Liang Liu, Hui Liu, Xiaodong Liu

**Affiliations:** ^1^Department of Orthopedics, Yangpu Hospital, Tongji University School of Medicine, Shanghai 200090, China; ^2^Center for Clinical Research and Translational Medicine, Yangpu Hospital, Tongji University School of Medicine, Shanghai 200090, China; ^3^Institute of Gastrointestinal Surgery and Translational Medicine, Tongji University School of Medicine, Shanghai 200090, China

## Abstract

Nucleus pulposus (NP) is the core substance to maintain the homeostasis of intervertebral disc and stability of biomechanics. The insufficient supply of nutrition (especially glucose) is an important factor that leads to the degeneration of NP cells. circRNAs play an important role in the process of intervertebral disc degeneration (IDD) by regulating the functions of NP cells. However, glucose deprivation-related circRNAs and their functions in IDD have not been reported. In this study, the differentially expressed circRNAs in NP cells after 0, 6, 12, and 24 h of glucose deprivation culture were detected by a microarray assay. Besides, time series clustering analysis by STEM software obtained the differentially up- and downregulated circRNAs during glucose deficiency. Then, the main functions and pathways of up- and downregulated circRNAs were predicted by the functional enrichment analysis. By constructing the circRNA-miRNA regulatory network, the potential mechanisms of the most differentially expressed circRNAs were predicted. In addition, according to in vitro validation, circ_0075062 was upregulated in degenerating NP tissues and glucose deprivation-induced NP cell degeneration. Based on Sanger sequencing and RNase tolerance assay, circ_0075062 was the circular transcript. Interfering with circ_0075062 expression could potentially alleviate the imbalance of extracellular matrix (ECM) synthesis and degradation in the NP cells induced by glucose deprivation. Together, these findings help us gain a comprehensive understanding of the underlying mechanisms of IDD, and circ_0075062 may be a promising therapeutic target of IDD.

## 1. Introduction

Intervertebral disc degeneration (IDD) is the main contributor to low back pain, which causes heavy economic burdens on society and families, as well as negative impacts on the quality of patients' work and life [1]. Multiple factors (such as heredity, environment, age, mechanics, and nutrition) and inflammatory factors (TNF, IL-1) are related to IDD [2–5]. However, the specific mechanism is still unknown. At present, medicine and operation are common ways of treating degenerative disc disease. But the success rate of relieving long-term pain is rather low, which may also induce abrasions among adjacent segments [6]. Hence, it is of high clinical significance to gain a deep understanding of the pathogenesis and pathological process of IDD, so as to seek therapeutic methods based on pathogeny.

Nucleus pulposus (NP) cells play an important role in maintaining the integrity of the intervertebral disc by secreting the extracellular matrix [7]. Abnormal NP cell function, such as reduced proliferation, cluster formation, and cellular senescence, can accelerate the development of IDD [8, 9]. The intervertebral disc is the largest nonvascular tissue in the human body. NP cells gain nutrition from the diffusion of capillaries in the endplate; thus, their nutrition supply is limited for long time [10]. Glucose is one of the nutritional ingredients for NP cells. The concentration gradient of glucose in adults' NP cells declines from 5.5 mM in endplates to 0.5 mM and even lower in the center of NP [11]. According to previous studies, the decreasing supply of nutrition, especially glucose, has a significant impact on the proliferation or survival of NP cells [12–14]. For this reason, further research and studies on the potential mechanism of NP cell dysfunction in the shortage of glucose may provide new strategies for preventing and treating IDD.

circRNAs are a new type of RNA with covalent and closed-loop structures and no 5′cap or 3′ tail. Due to the cyclic structures, circRNAs are not prone to the degradation of RNA exonuclease and more stable than linear RNA. Moreover, circRNAs demonstrate unique traits of illnesses and developmental stages in different pathological contexts, indicating that circRNAs have more advantages in serving as disease diagnosis and therapeutic targets [15, 16]. More and more evidence shows that circRNAs play important roles in IDD development [17–19]. However, glucose deprivation-related circRNAs and the functions in IDD have not been reported yet.

In this study, by microarray analysis and time series clustering analysis, we obtained up- and downregulated circRNAs in NP cells under glucose deprivation for 0, 6, 12, and 24 h. Then, the role of differentially expressed circRNAs in glucose deprivation-induced NP cell degeneration is investigated by bioinformatics prediction and in vitro validation. Our results contribute to the understanding of the underlying mechanisms of IDD and establish a novel research target for IDD.

## 2. Materials and Methods

### 2.1. Ethical Statement

This research program has been approved by the ethical review committee of Yangpu Hospital, Tongji University. We have acquired the informed consent form from all participants involved in the study.

### 2.2. Cell Culture and Treatment

Human NP cells were purchased from ScienCell Research Laboratories (Carlsbad, CA, USA), which had been extracted from nucleus pulposus. After being resuscitated, NP cells were cultured in DMEM/F12 with 10% fetal bovine serum and 100 mg/ml streptomycin at the temperature of 37°C and 5% CO_2_ humidity. The culture medium was replaced once every three days. The second-generation cells were used in subsequent experiments. In the complete medium with no glucose, nucleus pulposus cells were incubated for 0 h, 6 h, 12 h, and 24 h. Total RNA was extracted from NP cells at each time node with three biological repetitions.

### 2.3. RNA Extraction

Total RNA containing small RNA was extracted using the Trizol reagent (Invitrogen) and purified with the mirVana miRNA Isolation Kit (Ambion, Austin, TX, USA) according to the manufacturer's protocol. In addition, a spectrophotometer (NanoDrop ND-1000) was utilized to determine RNA purity and concentration from OD260/280 reading. RNA integrity was confirmed by 1% agarose gel electrophoresis.

### 2.4. circRNA Microarray Imaging and Data Analysis

The extracted RNA was digested, dephosphorylated, transformed, extended, and marked with Cy3-dCTP according to the manufacturer's protocol. The marked RNA was mixed with the microarray (CapitalBio Technology Human CircRNA Array v2) which also included circRNA probes from 170,304 persons. Then, the Agilent Scanner G2505C (Santa Clara, USA) was used in cleaning, fixating, and scanning the arrays to obtain images. Furthermore, GeneSpring software V13.0 (Agilent) summarized circRNA array data and analyzed standardization and quality control. In order to select differentially expressed circRNA, we adopted threshold values ≥ 2 and ≤−2. The Benjamini-Hochberg revised *p* value was 0.05. In addition, the Adjust Data function of the software CLUSTER 3.0 was used to conduct log_2_ conversion of data and confirm the midvalue-based centered upon genes. Lastly, further analysis was carried out using hierarchical clustering with average linkage.

### 2.5. Time Series Clustering Analysis

Short Time-series Expression Miner (STEM) software was used for data clustering analysis. This software program is specifically designed for analyzing short time series (3 to 8 temporal points) microarray gene expression data. The genes were allocated to a set of predefined model configuration files, so as to correctly detect temporal distribution with relevant associative functions [20, 21].

### 2.6. Function and Pathway Enrichment Analysis

Gene Ontology (GO) analysis [22] of predicting target genes and pathway analysis of Kyoto Encyclopedia of Genes and Genomes (KEGG) [23] were conducted through online tools. The database was used in annotation, visualization, and comprehensive discovery (DAVID, http://david.ncifcrf.gov/) [24]. GO analysis depicted our understanding of biology from three aspects: biological process (BP), cellular component (CC), and molecular function (MF). KEGG was the integrated database resource designed for explaining genomic sequence and other high-throughput data. *p* < 0.05 was regarded as the threshold value.

### 2.7. Construct a circRNA-miRNA Regulatory Network

According to the latest research, circRNA could be used as the miRNA sponge to regulate gene expressions. In order to assess the potential function of circRNA, we used the miRanda algorithm to predict the target miRNA (miRanda score ≥ 140) of differentially expressed circRNA [25]. Target miRNA capable of combining more than three candidate circRNAs was identified as reliable interaction pairs of circRNA-miRNA. Cytoscape software was used to visualize the circRNA-miRNA regulatory network.

### 2.8. Clinic Sample Collection

This study involved 30 cases of surgical specimens of patients with IDD from August 2018 to January 2020 in Yangpu Hospital, Tongji University, including 14 males and 16 females aged between 34 and 80, and the average age was 58.3 years old. Based on the Pfirrmann grading systems, IDD patients were divided into the control group (II-III) and the degeneration group (IV-V). The selected patients had no past records of tumor, operation, hypertension or diabetes, etc. The gathered NP tissue was used in qRT-PCR and structure verification for the candidate circRNA.

### 2.9. Sanger Sequencing and RNase R Processing

NP tissue RNA was extracted. According to the circRNA circularization site, specific divergent primers were designed. Then, TA cloning and sequencing were carried out on the amplified products of divergent primers.

Total RNA of NP tissues was extracted and processed at 37°C with or without RNase R to obtain cDNA through inverse transcription of arbitrary primers. Then, cDNA was amplified by convergent primers and divergent primers. Electrophoresis of the PCR product was carried out with 2% agarose gel electrophoresis.

### 2.10. Quantitative Real-Time PCR (qRT-PCR)

The Trizol reagent was used to extract total RNA of NP cells and NP tissues. RNA concentration, purity, and integrity were detected by ND-1000 spectrophotometry and 1% agarose gel electrophoresis. Based on the instructions of PrimeScript™ RT Reagent Kit with Genomic DNA (gDNA) Eraser Kit, complementary DNA (cDNA) was synthesized through reverse transcription. qRT-PCR was carried out based on instructions of SYBR Premix Ex Taq II kits. 18s was the internal reference of circRNA. The relative expression level of data was analyzed by the standard curve method. The specific primers used are listed in Table 1.

### 2.11. Western Blotting

Cells were transfected with the circ_0075062 interference plasmid using Lipofectamine 2000 (Invitrogen, Carlsbad, CA, USA), according to the manufacturer's protocol. Then, transfected cells were collected for western blotting analysis after 24 h of glucose deprivation incubation. Briefly, treated NP cells were completely lysed in ice-cold RIPA Lysis Buffer containing 1% Protease Inhibitor Cocktail (Sigma-Aldrich) and then centrifuged at 12000 r/min for 15 min. The bicinchoninic acid protein assay was performed to monitor the protein concentration. Then, proteins were separated in a 10% SDS-PAGE gel and transferred to PVDF membranes. The membranes were subsequently blocked with 3% bovine serum albumin/Tris-buffered saline containing Tween 20 for 2 hours and then incubated with anti-MMP-13 (Affinity, AF5355, 1 : 1000), anti-aggrecan (Affinity, DF7561, 1 : 1000), anti-collagen II (Affinity, AF0135, 1 : 1000), and anti-GAPDH (Sangon, D110016, 1 : 1000) at 4°C overnight and then incubated with a 1 : 5000 secondary antibody for 2 hours. The protein signals were detected by the enhanced chemiluminescence method. GAPDH was used as an endogenous normalization of protein. The intensities of bands were analyzed using ImageJ.

### 2.12. Statistical Analysis

Statistical analysis was conducted with GraphPad Prism 8.0. All data were represented by means ± SEM. Each group of the experiment was repeated three times. Student's *t*-test or one-way analysis of variance was used to analyze the significance of differences. *p* < 0.05 was regarded as having statistical significance.

## 3. Results

### 3.1. circRNA Expression Profile Analysis in Glucose Deprivation-Induced NP Cell Degeneration

**Figure 1:**
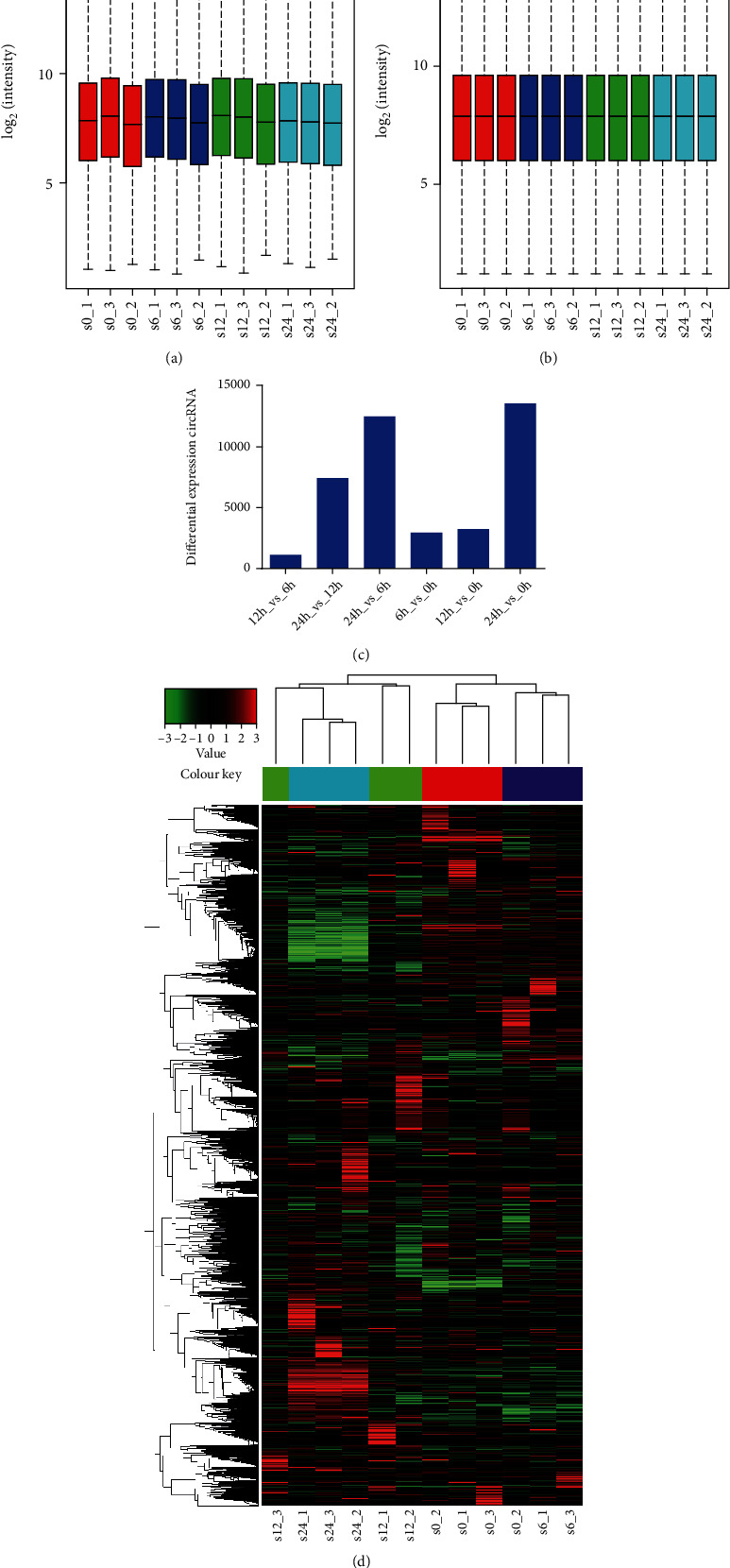
Expression profiles of circRNAs in NP cells during glucose deprivation. (a, b) The box plot showing the distribution of circRNA profiles in 12 samples. After normalization, the distributions of log_2_ ratios among 12 samples are nearly the same (s0, s6, s12, and s24 represent that NP cells were incubated under glucose deprivation for 0, 6, 12, and 24 h, respectively). (c) The number of DECs identified after intercomparison at different time points. (d) Hierarchical clustering of all DECs in NP cells after being incubated under glucose deprivation for 0, 6, 12, and 24 h. DECs: differentially expressed circRNAs.

In order to find out the potential role of circRNAs in glucose deprivation-induced NP cell degeneration, the NP cells were incubated in the complete medium with no glucose for 0 h, 6 h, 12 h, and 24 h. Then, high-throughput circRNA microarray assays were performed. The expression value of microarray data before pretreatment is shown in Figure 1(a). Standardized medians were on the same horizontal line with a favorable effect (Figure 1(b)). Principal component analysis (PCA) is used to visualize clusters in 12 samples (Figure s1). *p* ≤ 0.05 and ∣log_2_(fold change) | ≥2 were used as a significant cutoff criterion to conduct differential expression analyses with respect to each time point (0, 6, 12, and 24 h), and 1135 (12h_vs_6h), 7438 (24h_vs_12h), 12521 (24h_vs_6h), 2953 (6h_vs_0h), 3246 (12h_vs_0h), and 13578 (24h_vs_0h) differential expression circRNAs were obtained, respectively (Figures 1(c) and 1(d), Figure s2).

### 3.2. Time Series Clustering Analysis

**Figure 2:**
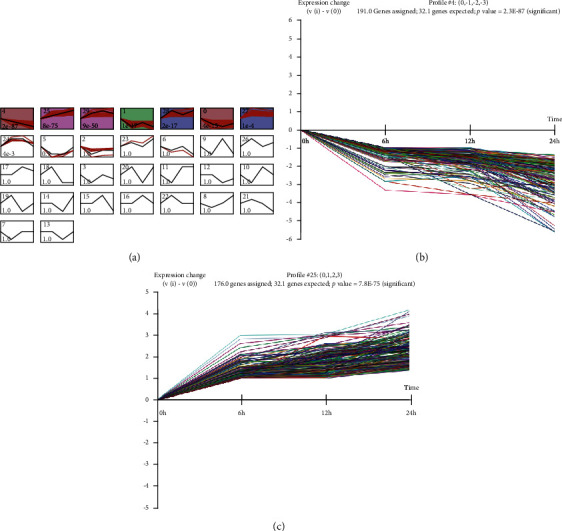
Clustering of time series circRNA expression data. (a) Profiles ordered based on the actual size-based *p* value of DECs. (b) Cluster that basically trended downward with the time points changed. (c) Clusters that basically trend upward with the time points changed.

The STEM software was used to conduct time series cluster analysis and obtain 30 circRNA clusters. Clustering results varied with time tendency (Figure 2(a)). Clusters with continuously increasing or declining trends as the differentially expressed circRNAs (DECs) for subsequent analysis are shown in Figures 2(b) and 2(c). Cluster 4 showed the declining trend, and Cluster 25 showed the upward trend, while other clusters indicated relatively complex changes without significant trends. Hence, we used Cluster 4 as the downregulated circRNAs as in the process of glucose deprivation and Cluster 25 as the upregulated circRNAs (Supplementary Files 1 and 2).

### 3.3. Functional Enrichment Analysis of DECs

**Figure 3:**
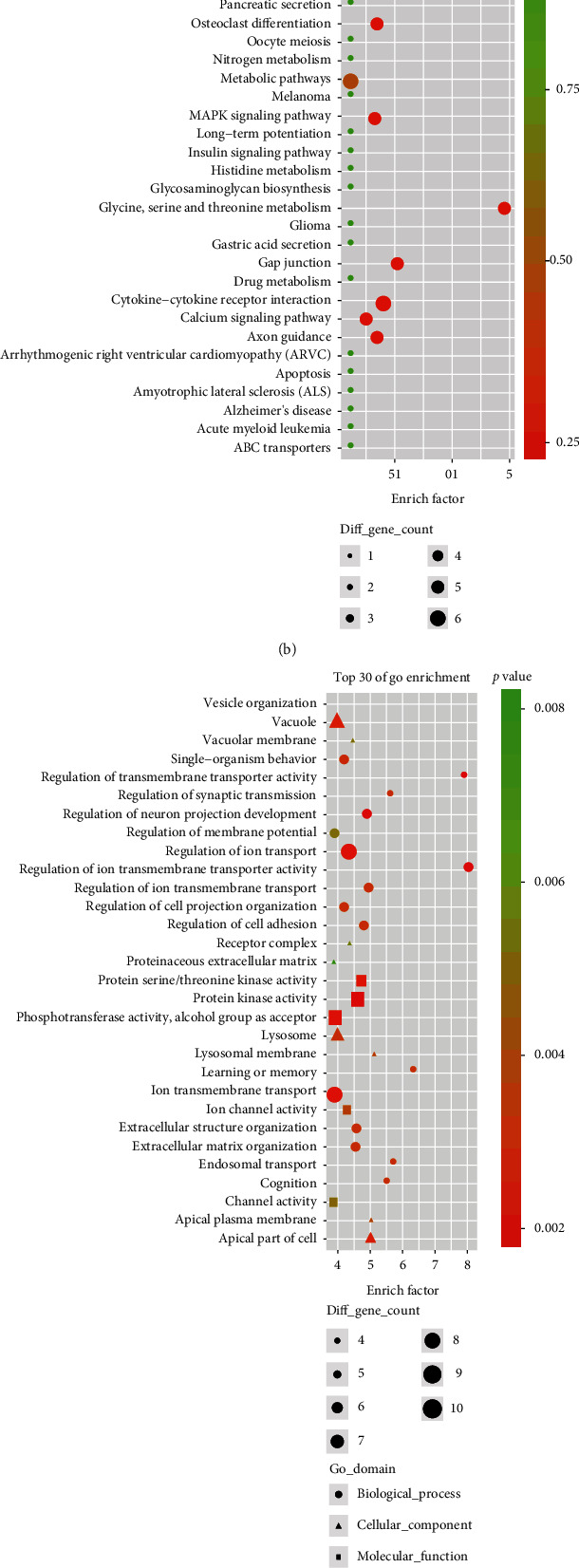
Functional enrichment analysis of up- and downregulated circRNAs. (a, b) Top 30 terms of GO and KEGG pathway enrichment of downregulated circRNAs. (c, d) Top 30 terms of GO and KEGG pathway enrichment of upregulated circRNAs. The circles represent biological process, the triangles represent cell component, and the squares represent molecular function.

For host genes of the selected up- and downregulated circRNAs, GO and KEGG pathway enrichment analyses were performed to predict their potential functions. The top 30 GO and pathway items are shown in the bubble diagram (Figure 3). We found that the gene symbol of downregulated circRNAs is mainly enriched in biological processes such as cytoskeleton organization and cell migration, as well as pathways such as cytokine-cytokine receptor interaction and metabolic pathways (Figures 3(a) and 3(b)). Functions of the gene symbol in upregulated circRNAs are enriched in biological processes like ion transmembrane transport and regulation of ion transport (Figure 3(c)) and pathways like focal adhesion, ECM-receptor interaction, and calcium signaling pathway (Figure 3(d)).

### 3.4. circRNA-miRNA Regulatory Network Construction

**Figure 4:**
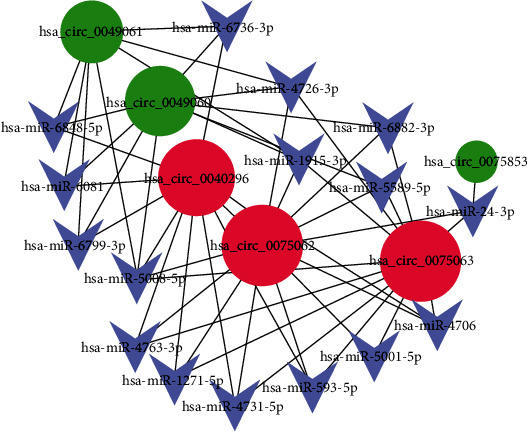
Construction of the circRNA-miRNA regulatory network (the number of miRNAs regulating circRNAs in this network should be ≥3). Red circle and green circle represent up- and downregulated circRNAs, respectively. Blue arrow represents target miRNA.

Based on the miRanda algorithm, the target miRNAs of the top 5 up- and downregulated circRNAs (Table 2) were predicted. As we all know, one type of miRNA could combine with one or more circRNAs. We selected miRNA that could combine with more than three circRNAs to construct the circRNA-miRNA regulatory network, as shown in Figure 4. circ_0075062 (degree, 12) and circ_0075063 (degree, 12) were two upregulated circRNAs with the highest connectivity. Besides, miR-5008-5p (degree, 5), miR-1915-3p (degree, 4), and miR-4726-3p (degree, 4) were the target miRNAs with high connectivity. These circRNAs and miRNAs may be the important regulator in glucose deprivation-induced NP cell degeneration.

### 3.5. Expression and Characterization of circ_0075062

**Figure 5:**
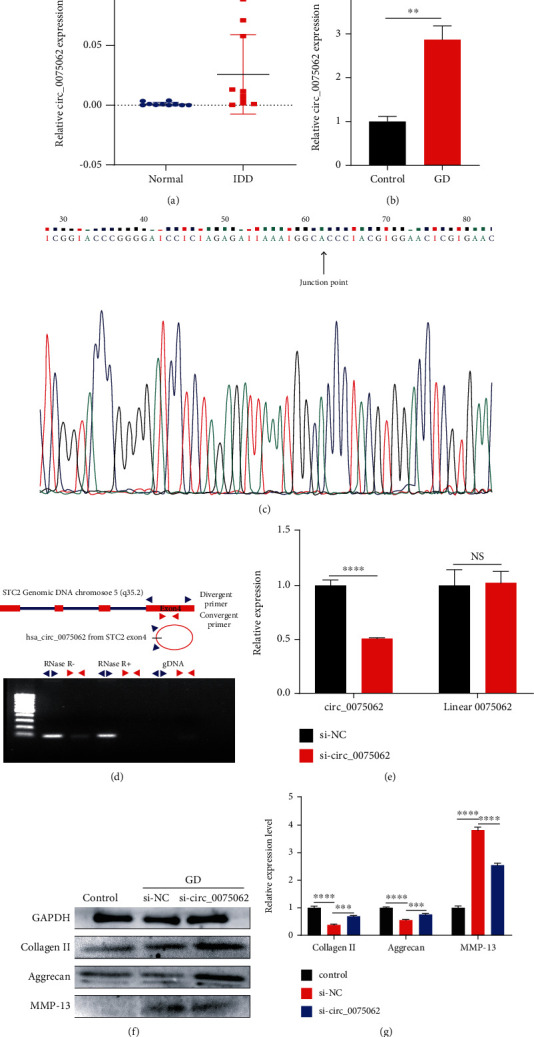
Expression and characterization of circ_0075062. (a, b) The expression level of circ_0075062 in degenerated NP tissues and NP cells cultured under glucose deprivation was detected by qRT-PCR. (c) Sanger sequencing of circ_0075062 showed the back-splice junction. (d) circ_0075062 is derived from exon 4 of the STC2 gene. Divergent primer detected circ_0075062 in cDNA but not gDNA. (e) The relative expressions of circ_0075062 and linear 0075062 in NP cells were determined by qRT-PCR after transfecting with the circ_0075062 interference plasmid. (f, g) After transfecting with the circ_0075062 interference plasmid, the protein expression level of collagen II, aggrecan, and MMP-13 was analyzed with western blotting under glucose deprivation for 24 h. NS: not significant; GD: glucose deprivation. ^∗^*p* < 0.05, ^∗∗^*p* < 0.01, ^∗∗∗^*p* < 0.001, and ^∗∗∗∗^*p* < 0.0001.

In vitro validation was performed on the selected key circRNAs. qRT-PCR results showed that circ_0075062 apparently increased in the NP cells under glucose deprivation condition and degenerative NP tissues, which were in accordance with microarray data (Figures 5(a) and 5(b)). Sanger sequencing was used to verify PCR amplified product, which indicated that circ_0075062 was head-to-tail splicing (Figure 5(c)). circ_0075062 originated from the fourth exon of STC2 genes. Due to the special topological structures, circRNA was quite resistant to RNase R. After being digested by RNase R, the treatment group could still detect circ_0075062, but linear RNA could not be detected (Figure 5(d)). The above results showed that circ_0075062 was a circular transcript. To evaluate the specificity of the circ_0075062 interference plasmid targeting the circular structure of circ_0075062, we determined the relative expression of circ_0075062 and linear 0075062 in NP cells (Figure 5(e)). The results showed that the circ_0075062 interference plasmid downregulated the expression of circ_0075062, while the expression of linear 0075062 was not affected, indicating successful circ_0075062 interference. Downregulation of circ_0075062 promotes ECM synthesis (collagen II and aggrecan) and inhibits ECM degradation (MMP-13) under glucose-restricted condition. Collectively, these results suggest that circ_0075062 is a novel target and downregulation of circ_0075062 can inhibit glucose deprivation-induced NP cell degeneration.

## 4. Discussions

IDD has always been a health issue around the world, which has brought heavy burdens to the healthcare system and the economy [26]. NP, as an important part of intervertebral disc, plays a critical role in maintaining the microenvironmental stability and mechanical property of the intervertebral disc. A comprehensive understanding of the NP dysfunction mechanism will lay the foundation for the recovery, regeneration, and tissue engineering strategies of IDD-related disease [27].

circRNAs are a new type of ncRNA, which play important roles in regulating genetic transcriptions and expressions. Recent studies showed that circRNAs can regulate the development and progression of IDD, including cell proliferation, apoptosis, ECM synthesis/degradation, and the generation of proinflammatory cytokine [28]. Inadequate supply of nutrition (especially glucose) is a primary obstacle in the regeneration of NP cells [12–14]. However, the expression and function of glucose deficiency-associated circRNAs in IDD remain unknown. In this study, we obtained circRNA expression profiles in response to glucose deficiency using microarray technology and time series clustering analysis in an experimental nutrient deprivation cell model. By using the tools of bioinformatics, biological functions and regulatory mechanisms of DECs were predicted. Further in vitro experiments suggest that circ_0075062 may be a new target for IDD intervention.

With the development of biotechnology, microarray analysis has become a powerful tool for characterizing many pathophysiological processes. Microarray technology has also identified many diagnostic markers and therapeutic targets in IDD [29, 30]. Exposure of NP cells to serum deprivation [31], oxidative stress [32], and pressure response [33] is an effective in vitro model to study the molecular mechanism of IDD. In rat NP cells with serum starvation, microarray analysis indicated that significant differences in the genetic expressions of cell cycle DNA damage checkpoints [31]. In the compressed human NP cells, circRNA microarray analysis and qRT-PCR revealed that circRNA-CIDN was significantly downregulated and overexpression of circRNA-CIDN inhibited compression-induced apoptosis and NP ECM degradation [33]. Our previous studies found that glucose deprivation induced time-dependent morphological changes, proteoglycan degradation, and apoptosis in NP cells [34]. More and more research demonstrates that the analysis of microarray time series data plays an important role in bioinformatics studies and biomedical applications. The STEM software can obtain models with statistical significance from short time series microarray experiments. Besides, it can also compare the datasets among various experiments, presenting its analysis of the data in a highly visual and interactive manner [21]. Hence, our study can provide a more reliable and novel research target for IDD.

According to the GO analysis, downregulated circRNAs were mainly related to biological processes including cytoskeleton organization and cell migration. Upregulated circRNAs were related to biological processes including ion transmembrane transport and regulation of ion transport. IVD involves three cytoskeleton elements (F-actin, *β*-microtubulin, and vimentin), which are of significance in cell division, movement, protein transport, and secretion. F-actin can regulate the mechanical conduction between ECM and IVD. Cytoskeleton network disorder may contribute to the loss of IVD stability and catabolism phenotype [35]. Recently, Zhang et al. found that SM/J early degeneration of the intervertebral disc was related to changes in genetic expressions of the ion transport system [36]. Transient receptor potential (TRP) channels, a superfamily of multimodal ion channels, were perhaps the potential trigger for intervertebral disc disease. Sadowska et al. confirmed the significance of TRP channels in IVD stability and pathology, which may consider them pharmacological targets for the treatment of IDD and low back pain [37]. These evidences indicate that DECs may be involved in the pathophysiological processes of IDD by regulating the above biological processes. KEGG pathway analysis indicated that downregulated circRNAs were involved in cytokine-cytokine receptor interaction and metabolic pathways, while upregulated circRNAs were involved in focal adhesion and ECM-receptor interaction signal channels, which are closely related to NP cell regeneration, extracellular matrix synthesis, and catabolism [5, 38, 39]. Nutrition deprivation could lead to NP cell apoptosis and reduce the expression of collagen II and aggrecan, which are two basic and important elements in the stability of the intervertebral disc [31, 34]. Hence, we predicted that DECs may play important roles in IDD progression by regulating ECM metabolism.

circRNA can act as a sponge for miRNA, thus influencing its activity and increasing expression of miRNA target genes [40]. Cui and Zhang showed that circ_001653 combines with miR-486-3p to regulate biological characteristics of NP cells and ECM synthesis [18]. Guo et al. reported that circ-FAM169A enhanced ECM catabolism through the miR-583/BTRC axis, thereby promoting IDD progression [19]. An increasing number of circRNAs regulating the progression of IDD have been discovered. However, circRNA-mediated regulatory mechanisms are still poorly understood. In this study, by constructing the circRNA-miRNA regulatory network, we screened key circRNAs as well as important target miRNAs during glucose deprivation-induced NP cell degeneration. Further in vitro validation showed that circ_0075062 is involved in the ECM metabolic process of the NP cells under glucose deprivation condition, which is consistent with our bioinformatics predictions.

## 5. Conclusions

We explored the changes in the expression of circRNAs in NP cells during glucose deprivation. Through bioinformatics analysis and in vitro validation, circ_0075062 was identified as the key circRNA and predicted the potential mechanism of its regulation of IDD progression. This study enhanced the understanding of the molecular mechanism of IDD. The novel circ_0075062 may be the potential therapeutic target for IDD.

## Figures and Tables

**Table 1:** Sequences of primers used for qRT-PCR.

Name	Sequence	Product size (bp)
circ_0075062	F: AAATGGCACCCTACGTGGAC	107
	R: TCCCCAGTTCTGCTCACACT	

18S	F: AGTCGCCGTGCCTACCAT	129
	R: CGGGTCGGGAGTGGGTAAT	

Divergent	F: AAATGGCACCCTACGTGGAC	107
	R: TCCCCAGTTCTGCTCACACT	

Convergent	F: TATCAAACAGCGACATAGCCATAC	100
	R: ACTCCCCAGGTGTGTATTTTCC	

**Table 2:** Top 5 upregulated and downregulated circRNAs in glucose deprivation-induced degenerative NP cells.

circRNA	*p* value (0, 6, 12, and 24 h)	Regulation	Chromosome	Strand	Gene symbol
hsa_circ_0075063	0, 2.98, 3.04, 4.16	Up	chr5	−	STC2
hsa_circ_0134113	0, 1.34, 1.62, 4.09	Up	chr7	−	PDE1C
hsa_circ_0040296	0, 1.98, 2.87, 3.98	Up	chr16	+	IL34
hsa_circ_0075062	0, 2.82, 2.92, 3.89	Up	chr5	−	STC2
hsa_circ_0133907	0, 2.63, 3.02, 3.57	Up	chr7	+	HDAC9
hsa_circ_0131556	0, −1.3, −1.84, −5.4	Down	chr6	−	FAM65B
hsa_circ_0075853	0, −1.68, −1.89, −5.3	Down	chr6	−	FAM65B
hsa_circ_0049060	0, −1.46, −2.34, −4.82	Down	chr19	+	ANGPTL4
hsa-circ_14617-6	0, −1.13, −1.83, −4.81	Down	chr6	−	FAM65B
hsa_circ_0049061	0, −1.13, −2.29, −4.7	Down	chr19	+	ANGPTL4

## Data Availability

The microarray datasets used and/or analyzed in this study are available from the corresponding author on reasonable request.

## Abbreviations

circRNAs: Circular RNAs
NP: Nucleus pulposus
IDD: Intervertebral disc degeneration
DMEM: Dulbecco's modified Eagle's medium
STEM: Short Time-series Expression Miner
GO: Gene Ontology
KEGG: Kyoto Encyclopedia of Genes and Genomes
qRT-PCR: Quantitative real-time PCR
gDNA: Genomic DNA
cDNA: Complementary DNA
PCA: Principal component analysis
DECs: Differentially expressed circRNAs
STC2: Stanniocalcin 2
ECM: Extracellular matrix.

## Supplementary Materials

Supplementary Materials Figure s1: principal component analysis of 12 samples. Figure s2: volcano plot of the DECs after intercomparison at different time points. Supplementary File 1: downregulated circRNAs in NP cells during glucose deprivation. Supplementary File 2: upregulated circRNAs in NP cells during glucose deprivation.

## References

[1] Gore M., Sadosky A., Stacey B. R., Tai K. S., Leslie D. (2012). The burden of chronic low back pain: clinical comorbidities, treatment patterns, and health care costs in usual care settings. *Spine (Phila Pa 1976)*.

[2] Feng Y., Egan B., Wang J. (2016). Genetic factors in intervertebral disc degeneration. *Genes Dis*.

[3] Vo N. V., Hartman R. A., Patil P. R. (2016). Molecular mechanisms of biological aging in intervertebral discs. *Journal of Orthopaedic Research*.

[4] Giers M. B., Munter B. T., Eyster K. J. (2017). Biomechanical and endplate effects on nutrient transport in the intervertebral disc. *World Neurosurgery*.

[5] Risbud M. V., Shapiro I. M. (2014). Role of cytokines in intervertebral disc degeneration: pain and disc content. *Nature Reviews Rheumatology*.

[6] Virk S. S., Niedermeier S., Yu E., Khan S. N. (2014). Adjacent segment disease. *Orthopedics*.

[7] Hughes S. P., Freemont A. J., Hukins D. W. L., McGregor A. H., Roberts S. (2012). The pathogenesis of degeneration of the intervertebral disc and emerging therapies in the management of back pain. *The Journal of Bone and Joint Surgery. British volume*.

[8] Johnson W. E., Eisenstein S. M., Roberts S. (2001). Cell cluster formation in degenerate lumbar intervertebral discs is associated with increased disc cell proliferation. *Connective Tissue Research*.

[9] Feng C., Liu H., Yang M., Zhang Y., Huang B., Zhou Y. (2016). Disc cell senescence in intervertebral disc degeneration: causes and molecular pathways. *Cell Cycle*.

[10] Guehring T., Wilde G., Sumner M. (2009). Notochordal intervertebral disc cells: sensitivity to nutrient deprivation. *Arthritis and Rheumatism*.

[11] Urban J. P., Smith S., Fairbank J. C. T. (2004). Nutrition of the intervertebral disc. *Spine*.

[12] Bibby S. R., Urban J. P. G. (2004). Effect of nutrient deprivation on the viability of intervertebral disc cells. *European Spine Journal*.

[13] Johnson W. E., Stephan S., Roberts S. (2008). The influence of serum, glucose and oxygen on intervertebral disc cell growth in vitro: implications for degenerative disc disease. *Arthritis Research & Therapy*.

[14] Saggese T., Thambyah A., Wade K., McGlashan S. R. (2020). Differential response of bovine mature nucleus pulposus and notochordal cells to hydrostatic pressure and glucose restriction. *Cartilage*.

[15] Chen L. L. (2016). The biogenesis and emerging roles of circular RNAs. *Nature Reviews. Molecular Cell Biology*.

[16] Patop I. L., Wüst S., Kadener S. (2019). Past, present, and future of circRNAs. *The EMBO Journal*.

[17] Cheng X., Zhang L., Zhang K. (2018). Circular RNA VMA21 protects against intervertebral disc degeneration through targeting miR-200c and X linked inhibitor-of-apoptosis protein. *Annals of the Rheumatic Diseases*.

[18] Cui S., Zhang L. (2020). circ_001653 silencing promotes the proliferation and ECM synthesis of NPCs in IDD by downregulating miR-486-3p-mediated CEMIP. *Mol Ther Nucleic Acids*.

[19] Guo W., Mu K., Zhang B. (2020). The circular RNA FAM169A functions as a competitive endogenous RNA and regulates intervertebral disc degeneration by targeting miR-583 and BTRC. *Cell Death & Disease*.

[20] Ernst J., Nau G. J., Bar-Joseph Z. (2005). Clustering short time series gene expression data. *Bioinformatics*.

[21] Ernst J., Bar-Joseph Z. (2006). STEM: a tool for the analysis of short time series gene expression data. *BMC Bioinformatics*.

[22] Gene Ontology Consortium (2006). The Gene Ontology (GO) project in 2006. *Nucleic Acids Research*.

[23] Kanehisa M. (2002). The KEGG database. *Novartis Foundation Symposia*.

[24] Huang D. W., Sherman B. T., Lempicki R. A. (2009). Systematic and integrative analysis of large gene lists using DAVID bioinformatics resources. *Nature Protocols*.

[25] Betel D., Wilson M., Gabow A., Marks D. S., Sander C. (2008). The microRNA.org resource: targets and expression. *Nucleic Acids Research*.

[26] Martin B. I., Deyo R. A., Mirza S. K. (2008). Expenditures and health status among adults with back and neck problems. *JAMA*.

[27] Alkhatib B., Ban G. I., Williams S., Serra R. (2018). IVD development: nucleus pulposus development and sclerotome specification. *Curr Mol Biol Rep*.

[28] Li Z., Chen X., Xu D., Li S., Chan M. T. V., Wu W. K. K. (2019). Circular RNAs in nucleus pulposus cell function and intervertebral disc degeneration. *Cell Proliferation*.

[29] Wang Y., Dai G., Wang L. (2019). Identification of key genes potentially related to intervertebral disk degeneration by microarray analysis. *Genetic Testing and Molecular Biomarkers*.

[30] Liu X., Che L., Xie Y. K. (2015). Noncoding RNAs in human intervertebral disc degeneration: an integrated microarray study. *Genom Data*.

[31] Sudo H., Yamada K., Iwasaki K. (2013). Global identification of genes related to nutrient deficiency in intervertebral disc cells in an experimental nutrient deprivation model. *PLoS One*.

[32] Laboratory of Cell Proliferation and Ageing, Institute of Biosciences and Applications, National Centre for Scientific Research, Demokritos, 153 10 Athens, Greece (2015). Oxidative stress inhibits the proliferation, induces premature senescence and promotes a catabolic phenotype in human nucleus pulposus intervertebral disc cells. *European Cells & Materials*.

[33] Xiang Q., Kang L., Wang J. (2020). CircRNA-CIDN mitigated compression loading-induced damage in human nucleus pulposus cells via miR-34a-5p/SIRT1 axis. *eBioMedicine*.

[34] Chang H., Cai F., Zhang Y. (2017). Early-stage autophagy protects nucleus pulposus cells from glucose deprivation-induced degeneration via the p-eIF2*α*/ATF4 pathway. *Biomedicine & Pharmacotherapy*.

[35] Li S., Duance V. C., Blain E. J. (2007). F-actin cytoskeletal organization in intervertebral disc health and disease. *Biochemical Society Transactions*.

[36] Zhang Y., Xiong C., Kudelko M. (2018). Early onset of disc degeneration in SM/J mice is associated with changes in ion transport systems and fibrotic events. *Matrix Biology*.

[37] Sadowska A., Hitzl W., Karol A. (2019). Differential regulation of TRP channel gene and protein expression by intervertebral disc degeneration and back pain. *Scientific Reports*.

[38] Krouwels A., Melchels F. P. W., van Rijen M. H. P. (2018). Focal adhesion signaling affects regeneration by human nucleus pulposus cells in collagen- but not carbohydrate-based hydrogels. *Acta Biomaterialia*.

[39] Feng H., Danfelter M., Strömqvist B., Heinegård D. (2006). Extracellular matrix in disc degeneration. *The Journal of Bone and Joint Surgery. American Volume*.

[40] Hansen T. B., Jensen T. I., Clausen B. H. (2013). Natural RNA circles function as efficient microRNA sponges. *Nature*.

